# Visible light polarization-sensitive optical coherence tomography with balanced detection

**DOI:** 10.1117/1.JBO.30.3.036002

**Published:** 2025-03-11

**Authors:** Bahar Baradaran, Adam J. Black, Sarah R. Heilbronner, Taner Akkin

**Affiliations:** a University of Minnesota, Department of Biomedical Engineering, Minneapolis, Minnesota, United States; b Baylor College of Medicine, Department of Neurosurgery, Houston, Texas, United States

**Keywords:** axis orientation, birefringence, polarization-maintaining fiber, retardance

## Abstract

**Significance:**

We introduce a visible-light polarization-sensitive optical coherence tomography (PS-OCT) system that operates in the spectral domain with balanced detection (BD) capability. While the BD improves the signal-to-noise ratio (SNR), the use of shorter wavelengths improves spatial resolution and birefringence sensitivity.

**Aim:**

We aim to implement a new optical design, characterize its performance, and investigate the imaging potential for biological tissues.

**Approach:**

The design utilizes a unique interferometer and a custom spectrometer that captures four highly aligned spectra with a single area/multi-line camera. Each pair of spectral lines is highly aligned, and their subtraction yields balanced detected spectra of the PS-OCT channels. The resulting channels provide multiple imaging contrasts.

**Results:**

We measured the axial resolution and quantified the BD performance within the imaging depth. We also used a variable retarder to characterize the phase retardance and optic axis orientation measurements. Imaging results demonstrate the expected improvements for biological tissue.

**Conclusions:**

We successfully implemented BD for a high-resolution visible-light PS-OCT. Improved SNR and birefringence sensitivity allow better delineation of birefringent structures in biological tissues. This opens up new opportunities in the biomedical imaging field, especially for resolving structures and fibers that exhibit birefringence.

## 1 Introduction

Optical coherence tomography (OCT) is a label-free imaging modality providing non-contact depth-resolved biological tissue images at high spatiotemporal resolution.^1^ Various OCT designs have been reported in the time domain and spectral/Fourier domain for a number of applications.^2^ Each design has unique advantages and limitations, including specialized versions of OCT that can image blood flow and tissue anisotropy.

Polarization-sensitive optical coherence tomography (PS-OCT), an advanced form of OCT, provides additional imaging contrasts, including phase retardance and axis orientation based on tissue anisotropy called birefringence.^3^^,^^4^ Many biological tissues are optically anisotropic, allowing to delineate structures that differ in their organization. Typically, OCT and PS-OCT systems operate in the near-infrared spectrum. The current availability of low-coherent broadband laser sources in the visible-light range enables high-resolution imaging as well as enhanced birefringence sensitivity.^5^^,^^6^ However, increased light scattering at shorter wavelengths and the relative intensity noise of supercontinuum lasers^7^ hinder deep tissue imaging. Although the imaging depth may be increased by applying refractive index matching-based optical clearing agents,^8^ OCT systems with balanced detection (BD) capability can enhance the signal-to-noise ratio (SNR) by intensity noise subtraction.^9^ As the advantages of spectral domain OCT systems have been previously reported,^10^^–^^12^ PS-OCT imaging in the spectral domain can be further improved by BD operation.

The BD technique doubles the signal intensity while suppressing the common noise, such as autocorrelation and auto-interference components, fixed patterns, and source noise. Implementing BD is relatively easy for photodetector-based techniques (e.g., time-domain OCT and swept-source OCT).^13^ On the other hand, BD operation for spectral-domain (SD)-OCT is not straightforward as it relies on camera-based detection. This is due to the fact that the differential operation must be achieved for each pixel (wavelength) of the optical spectrum. This can be realized by using two carefully calibrated/matched spectrometers.^14^^–^^17^ BD systems with a single camera (multi-line/area or line-scan) have also been developed.^18^^–^^22^ These include a design that encoded light in interferometer arms with polarization states to produce two highly aligned spectra for detection by an area camera.^19^ Alternatively, using a single line-scan camera, BD was reported with an optical switch and a time delay,^21^ as well as a system consisting of three optical fiber couplers to generate the two spectra.^22^

In this paper, we present a novel visible-light PS-OCT system with BD capability in SD. The system features a uniquely designed interferometer and an innovative custom-built spectrometer, enabling single-shot BD operation with a single camera. The camera outputs two pairs of spectral lines, each of which is subtracted digitally to form the BD channels of PS-OCT. The system is characterized with and without BD. Imaging performance on biological tissues is also presented. The results demonstrate the expected improvements in SNR and birefringence sensitivity.

## 2 Materials and Methods

### 2.1 System Description

**Fig. 1:**
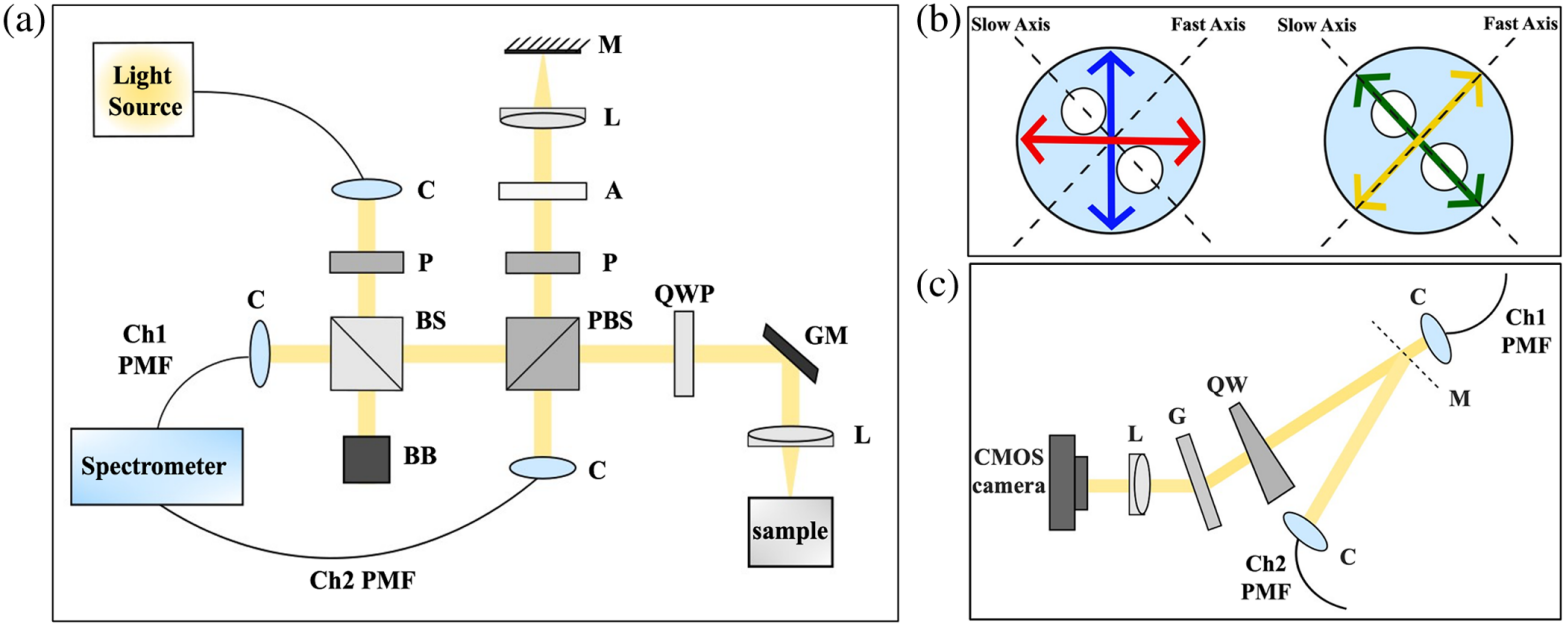
Schematics of the visible-light PS-OCT with BD. (a) Interferometer design. (b) Interference formation in the PM fiber; red and blue arrows indicate polarization states from reference and sample arms, and green and yellow arrows represent emerging BD channels. (c) Spectrometer design. (A attenuator, BB beam block, BS beamsplitter, C collimator, G grating, GM Galvo mirrors, L lens, M mirror, P polarizer, PBS polarizing beamsplitter, PMF PM fiber, QW quartz wedge, QWP quarter-wave plate).

The BD PS-OCT design, which has been recently described at an SPIE meeting,^23^ is demonstrated in Fig. 1(a). The light source is a supercontinuum laser (NKT Photonics, SuperK Fianium FIU-6 OCT) equipped with a bandpass filter (SuperK VARIA). Broadband light centered at 532 nm (spectral range: 137 nm between 467 and 604 nm) is fiber coupled to a multi-axis fiber bench (Thorlabs, FT-114X149) that houses achromatic fiberport collimators (Thorlabs, PAF2A-A15A) and other optical elements on fiberport mounts. This helps align the interferometer and couple the backscattered and reflected light to polarization-maintaining (PM) fibers, which transmit detected light to a custom-designed spectrometer. The power on the sample is 0.85 mW for tissue imaging, and the exposure time of the camera (Basler, acA2000-340kmNIR) is set to 24  μs, which is the minimum. The following sections present the design and operation of the system in detail.

#### 2.1.1 Interferometer

Incoming light from the source passes through a polarizer and a beamsplitter, which reflects ∼5% of the light toward the interferometer. On the way back, this beamsplitter transmits ∼95% of the returning light toward detection. The second beamsplitter is a polarization beamsplitter (PBS), which plays a key role in our interferometer design. The PBS transmits the vertical polarization state to the sample arm and reflects the horizontal polarization state to the reference arm. The splitting ratio of light for these arms is simply adjusted by rotating the initial polarizer.

The reference arm includes a polarizer at ∼45  deg, an attenuator, an achromatic focusing lens, and a reflector. The polarizer is adjusted to ensure that both PS-OCT channels receive equal amounts of back-reflected light from the reference arm. The sample arm comprises a quarter-wave plate (QWP), a two-axis galvanometer-based scanner, and a scan lens. As commonly used in PS-OCT configurations, the QWP at 45 deg converts linearly polarized light to circularly polarized light, illuminating the sample.^8^

Back-scattered and reflected light from the interferometer arms returns to the PBS, which diverts them to the detection paths. Because the polarization states of light from the reference and sample arms are linear and still orthogonal to each other, interference does not occur at this stage. Coupling light to the PM fibers is configured in a way that the fiber axes are 45 deg to these polarization states, as illustrated in Fig. 1(b). This is implemented by setting the ferrule connector/angled physical contact receptacle to nearly 45 deg and adjusting the fiber connector within the key to maximize the signal. Due to the propagation of low-coherent light in PM fiber, the projections carried in the fast and slow axes of the fiber become temporally separated and decorrelated. As a result, the projections of the sample and reference light in these axes share the same linear polarization states; therefore, the interference is formed when path lengths match. It is important to note that when constructive interference occurs in one PM fiber axis, destructive interference must occur in the other axis. This ensures the formation of BD channels (A and B) for each PS-OCT channel (Ch1 and Ch2). The BD channels are orthogonal, decorrelated, and carry out-of-phase signals.

#### 2.1.2 Spectrometer

Figure 1(c) shows the spectrometer design. Achromatic triplet lenses (f=40  mm) are used to collimate the light emerging from the PM fibers. A mirror, which is placed under the beam of Ch1, helps induce a small angular separation (∼1.7  deg) between the PS-OCT channels. Then, a 6.75 deg quartz wedge, whose axes are aligned with the PM fiber axes, splits the BD channels by introducing a slight angular separation (∼0.063  deg) between them. As a result, four angularly separated beams in the vertical direction enter a transmission grating (1800 lines per millimeter) at 29 deg. Consequently, an achromatic triplet lens (f=40  mm) focuses four lines of spectra on the area camera with 5.5  μm pixels.

### 2.2 Image Formation

In spectral domain systems, interference-related oscillations on the optical spectrum yield depth information.^24^ The spectral lines are interpolated to obtain spectra in linear k-space. Subtraction of spectral line pairs (A and B) is used to achieve BD. Then, dispersion mismatch is compensated in software.^25^ Finally, the inverse Fourier transform of Ch1 and Ch2 spectra yields complex-valued information along the depth (z) direction, $A_{1,2}(z)\exp(i\phi_{1,2}(z))$, where A and ϕ represent the amplitude and phase, and subscripts 1 and 2 correspond to the PS-OCT channels. The imaging contrasts are derived from the amplitude and phase information. These include reflectivity (R), phase retardance (δ), and optic axis orientation (θ), as formulated below.^26^

$$R(z)=A_{1}^{2}(z)+A_{2}^{2}(z),$$ (1)

$$\delta (z)=\arctan(\frac{A_{1}(z)}{A_{2}(z)}),$$ (2)

$$\theta (z)=(\pi -(\phi_{1}(z)-\phi_{2}(z)))/2.$$ (3)

In the sample arm, we utilized a pair of galvanometer-based scanners for raster scanning of the beam, which enables the construction of 2D or 3D images. A cross-sectional image (B-line) is formed by stacking multiple depth profiles (A-line) acquired while scanning the beam over the sample. The data can also be visualized with en-face images, which are the projections of A-lines onto the lateral plane.

## 3 Results

### 3.1 Creation of BD Channels

**Fig. 2:**
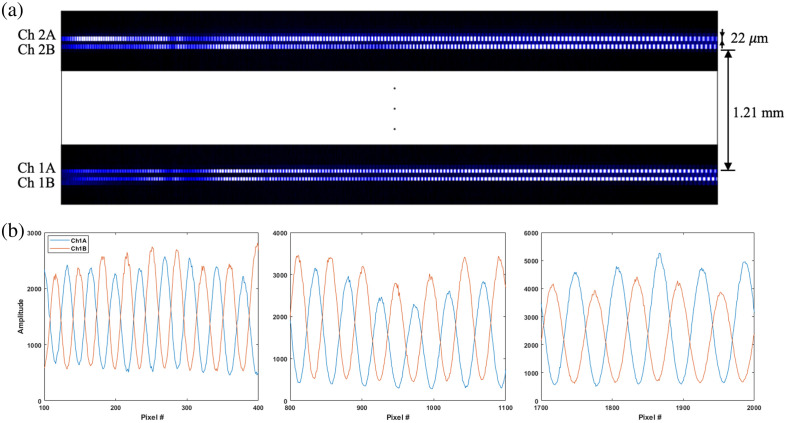
PS-OCT channels on the camera. (a) Spectral lines (pixel size: 5.5×22  μm; line length: 11.132 mm). (b) Oscillations due to interference maintain a 180-deg phase difference for the entire spectrum.

The spectral lines on the camera are shown in Fig. 2(a). Because the camera is configured with vertical binning of four pixels, each pixel is effectively rectangular with 5.5  μm width and 22  μm height. Closely separated lines (A and B) are 44  μm apart, and there is 1.21 mm between the two pairs of lines (PS-OCT channels). The alternating brightness along the lines represents spectral oscillations due to interference. For each channel, lines A and B are highly aligned, and their varying oscillation intensities in the vertical direction are due to the BD method. This is better demonstrated in Fig. 2(b), which plots pixel intensities in the left, middle, and right parts of Ch1A and Ch1B to indicate maintaining a 180-deg phase difference in the entire spectral range. The result is similar for Ch2 (not shown), though pixel-to-wavelength/wavenumber correspondence, which is highly aligned for closely separated lines, is not identical to those of Ch1.

### 3.2 Characterization

The system is characterized by placing a voltage-controlled liquid crystal variable retarder (LCVR, Meadowlark Optics) between the QWP and the lens in the sample arm. The combination of the LCVR and a partial reflector, which is a single glass-air interface (a wedged prism), mimics a birefringent sample. To prevent saturation of the camera pixels, the source power is reduced to ∼3%, which resulted in 19  μw power on the partial reflector with ∼4% reflection.

**Fig. 3:**
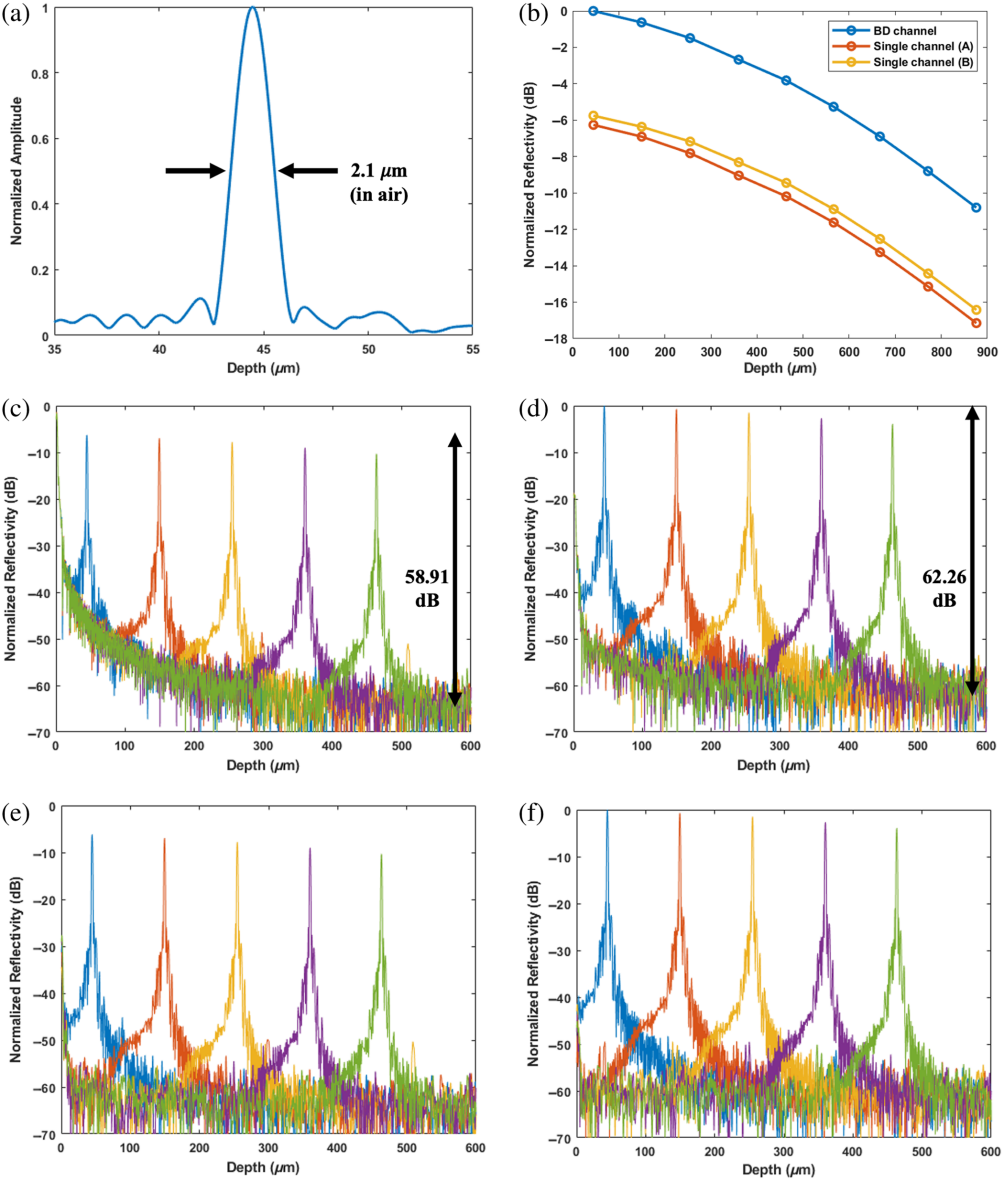
Amplitude measurements. Coherence function (a), BD signal gains over single channels (b), reflectivity profiles without (c) and with (d) BD, and background subtracted reflectivity profiles without (e) and with (f) BD.

The spectral lines are recorded at a constant LCVR voltage. Then, complex-valued depth profiles are calculated from the spectral data as described in Sec. 2.2. With 1024 pixels and 1.05  μm pixel size, each profile yields a maximum depth ($Z_{\max}$) of 1.075 mm in air. Figure 3(a) shows the coherence function of the system. The axial resolution, as measured from the full width at half maximum, is 2.1  μm in air. This corresponds to ∼1.5  μm resolution for tissue imaging (with an index of refraction of 1.4). Without BD operation, the coherence functions generated from channels A or B will have a similar shape but with a reduced amplitude.

To study the signal increase by BD, data are acquired for multiple depth locations. Figure 3(b) represents the reflectivity peak values with and without BD operation. The values are normalized by the BD peak at ∼45  μm. Considering the values at all depths, the mean and standard deviation of the BD improvement over single channels A and B are 6.33±0.04  dB and 5.66±0.05  dB, respectively. The amplitude difference between single channels A and B is likely due to imperfect efficiencies of optical components and slight alignment errors. More importantly, the improvement is consistent throughout the entire depth range. For instance, the data values at the deepest peak (876  μm) show an average BD improvement of 5.97 dB over the single channels, which is only 0.03 dB less than the theoretical value expected from doubling the signal amplitude.

Figure 3 also displays the normalized reflectivity profiles with and without BD operation for five measurements at various depths. The BD operation effectively suppresses the peak at zero and the subsequent decay. The background stabilizes much earlier for the BD traces. Compared with the noise levels measured between 550 and 600  μm the reflectivity peaks at ∼45-μm depth yield an SNR of 58.91 dB for single channel A and 62.26 dB for the BD signal [Figs. 3(c) and 3(d)]. The peak at zero and subsequent decay can be further suppressed by subtracting the background spectra, which accounts for independent reflections from the interferometer arms. Then, the computed depth profiles contain the signal peaks and the remaining noise, as demonstrated in Figs. 3(e) and 3(f) for single channel A and BD. In addition to the SNR improvement, another advantage of the BD system is the removal of the auto-interference of the sample reflections, which could be problematic for imaging high-scattering tissues (see Fig. 5).

**Fig. 4:**
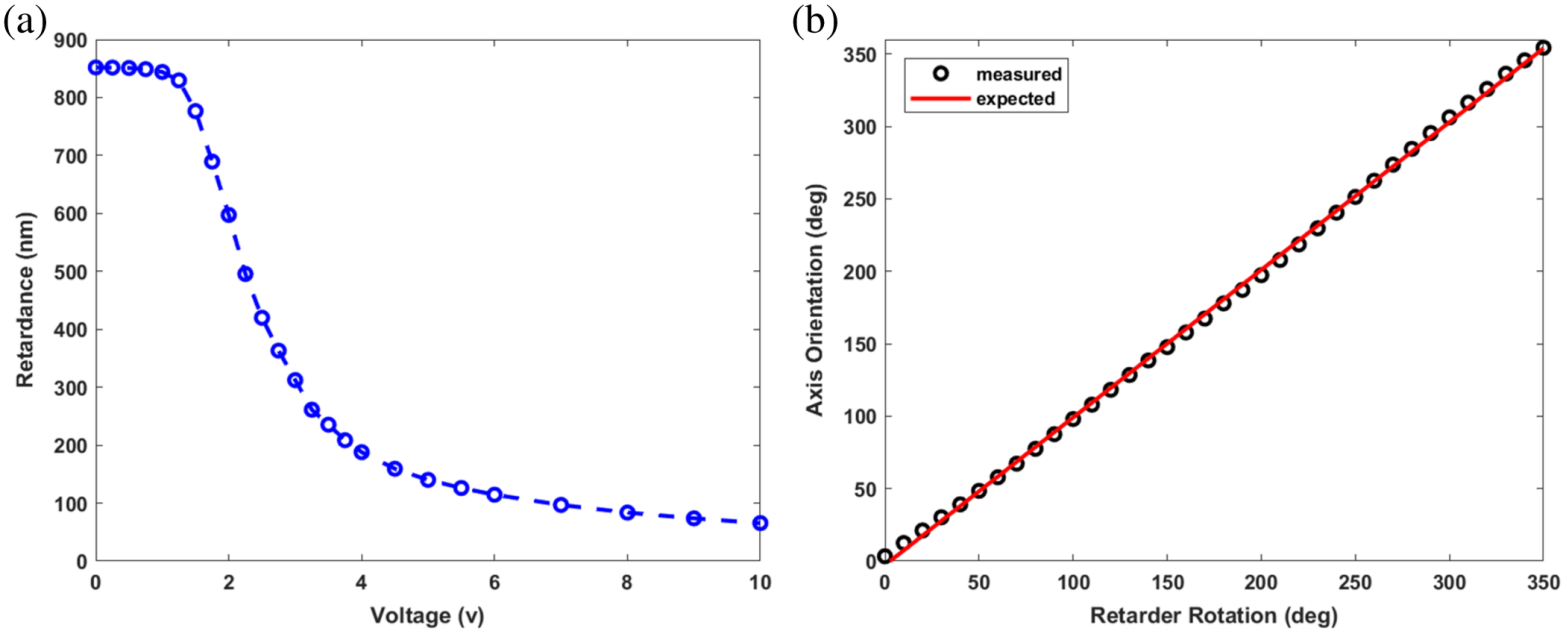
Retardance (a) and optic axis orientation (b) measurements from an LCVR.

To characterize the polarization contrasts, data are recorded from a fixed depth location for different voltage amplitudes that are applied to the LCVR. The resulting phase retardance values are calculated using Eq. (2). Figure 4(a) shows the unwrapped retardance values that are accumulated when birefringence is increased by lowering the LCVR voltage. The resulting shape of the curve is consistent with the manufacturer’s device specifications. For measuring optic axis orientation, the LCVR is rotated a full turn in steps of 10 deg, whereas its applied voltage is maintained at 10 V. Optic axis orientation values are calculated by using Eq. (3), and a fixed offset is subtracted to match the measured orientation with the optic axis of the retarder. Figure 4(b) presents the measured orientations (black circles) with respect to the retarder rotation. The measurements closely follow a line with a predicted slope of 1.

### 3.3 Biological Tissue Imaging

The imaging performance of the visible light PS-OCT system is evaluated by using various birefringent tissue samples, including rat tail tendon, leg muscle, and mouse brain. Rat tissue samples are harvested from a freshly sacrificed animal, and the skin is removed to expose the internal structures. The mouse brain sample is a slice from a fixed brain. All samples are imaged in PBS. A f=39  mm scan lens (LSM03-VIS) focuses the light on a sample. The power on the sample is 0.85 mW, and the spot size is ∼11  μm. The exposure time of the camera is set to 24  μs. Each cross-sectional image contains 800 A-lines.

**Fig. 5:**
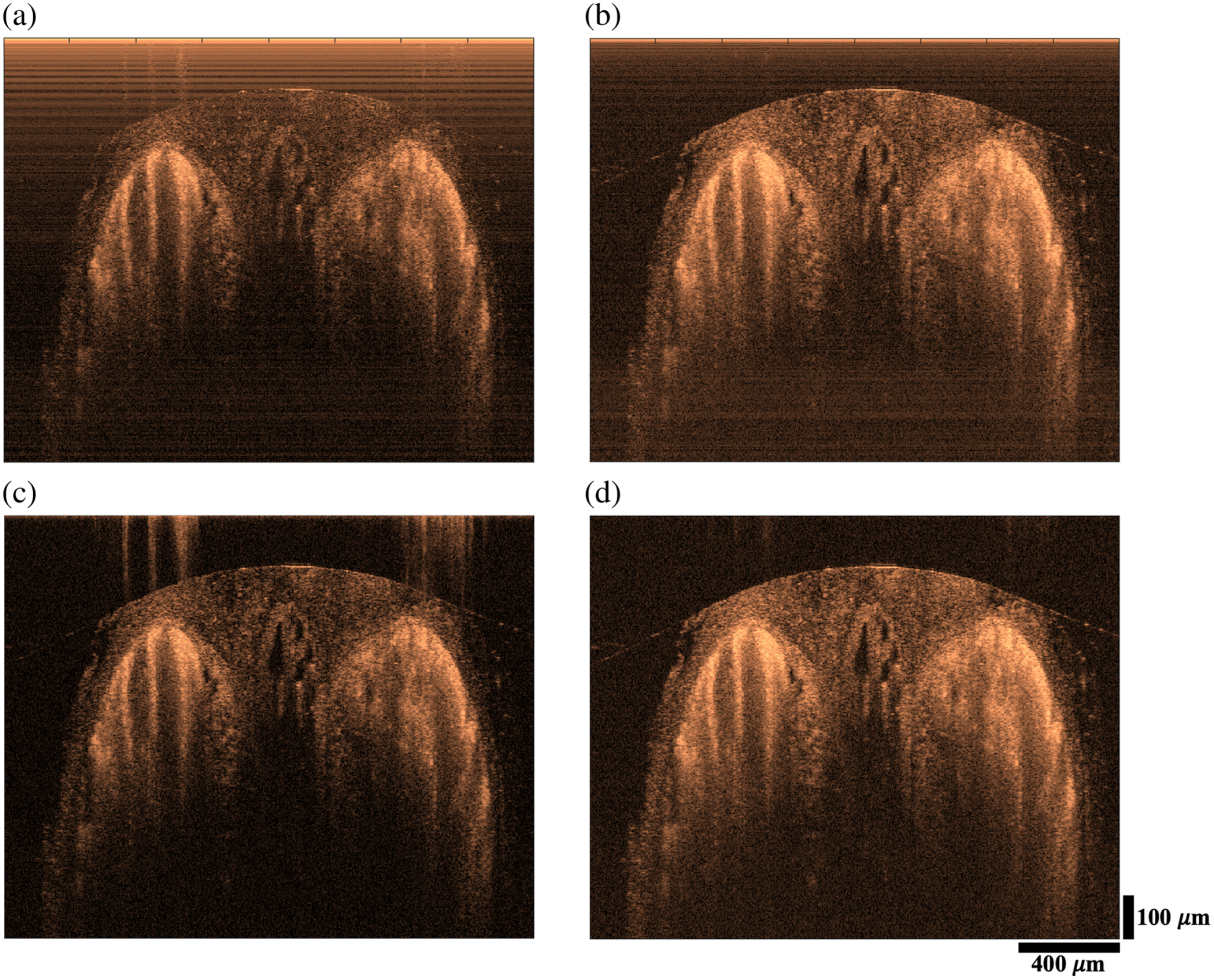
Cross-sectional reflectivity images of a rat tail tendon without BD (a), with BD (b), with background subtraction (c), and with BD and background subtraction (d). Dynamic range: 45 dB. Image size: 2.1 mm (x), 945  μm (z).

Figure 5 illustrates the reflectivity images acquired from a rat tail. High scattering within this sample contributes to a high dynamic range of 45 dB. The image in Fig. 5(a) is constructed from channel A without BD operation. Correspondingly, auto-interference of the sample reflections and autocorrelation of the source spectrum adversely impact the image quality. These artifacts are more problematic in the shallower regions that are known to be more sensitive in spectral domain imaging. Figure 5(b) shows the imaging result with BD operation. Importantly, the artifacts are effectively removed, and the visibility of structures is increased due to doubling the signal amplitude. As demonstrated in Fig. 5(c), background subtraction minimizes autocorrelation of the source spectrum and fixed pattern noise, but it does not affect the artifacts originating from the auto-interference of the sample reflections. As depicted in Fig. 5(d), the combination of the BD operation and background subtraction yields the highest-quality image characterized by greater brightness and reduced noise.

**Fig. 6:**
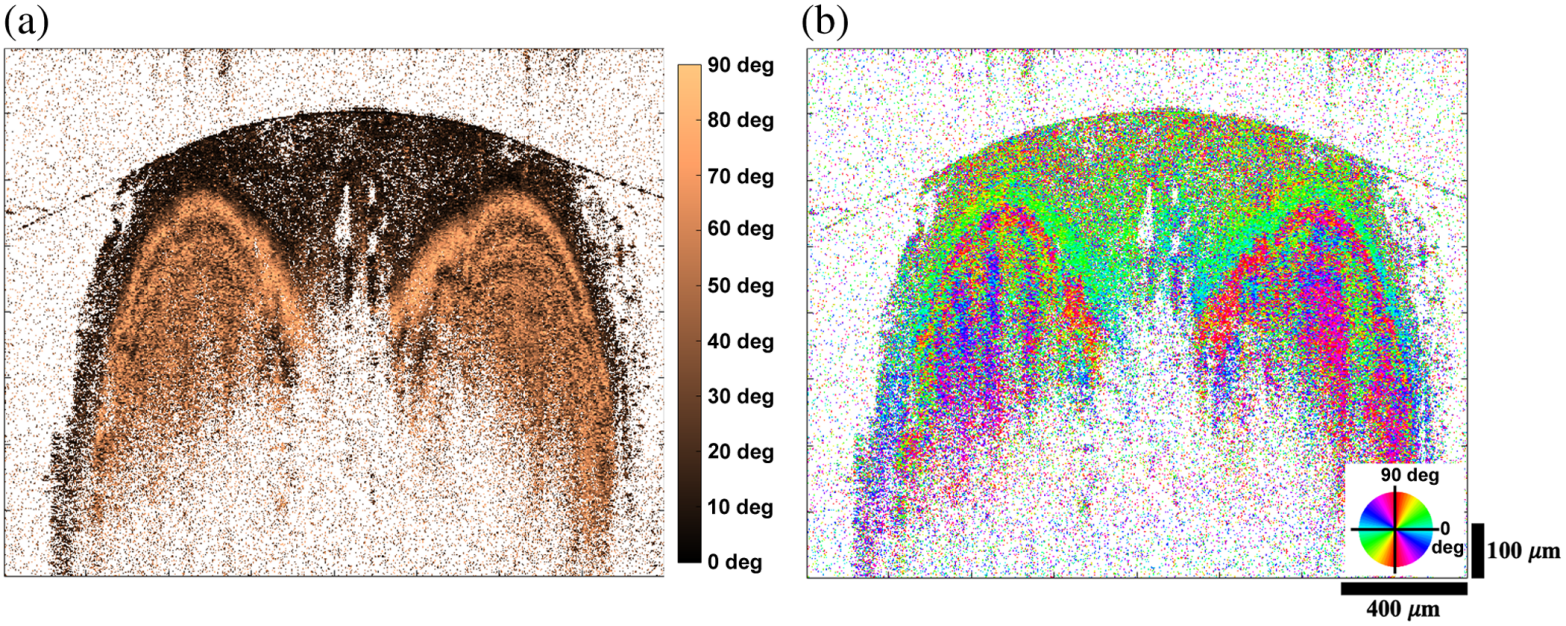
Phase retardance (a) and axis orientation (b) cross-sectional images of a rat tail tendon. BD operation, background subtraction, and thresholding are applied to enhance the visualization.

Figure 6 presents the birefringence images derived from the PS-OCT data of the same cross-section. The white background represents pixels with low reflectivity that are masked by a threshold. Tendon, as a highly birefringent tissue, alters the polarization state, which results in the accumulation of phase retardance. Because the phase retardance measurement is confined to a range of 0 deg to 90 deg, banding patterns in the depth direction occur, as shown in Fig. 6(a). Conversely, non-birefringent structures appear in a dark brown color. Figure 6(b) shows the optic axis orientation image with the +/−90  deg range. A wide variation of orientations is observed for non-birefringent regions. This is due to unreliable phase information of the cross-polarization channel whose amplitude is at the noise level. On the other hand, pixels for the highly birefringent tendon exhibit two distinct and alternating colors that are orthogonal. This behavior on the axis orientation occurs when the phase retardance exceeds 90 deg.

**Fig. 7:**
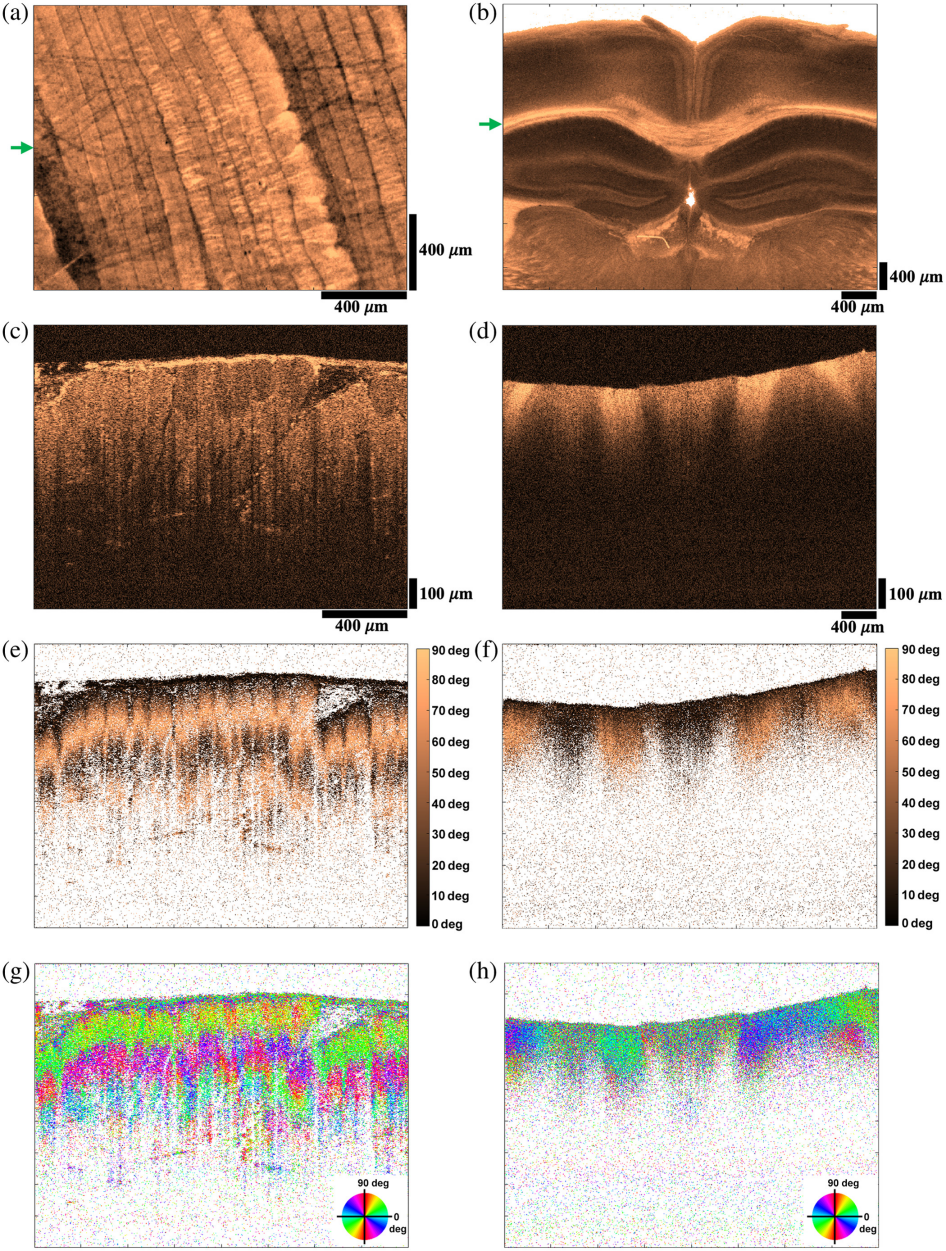
Different biological samples. En-face retardance images of rat muscle (a) and mouse brain (b), cross-sectional reflectivity images of rat muscle (c) and mouse brain (d), cross-sectional retardance images of rat muscle (e) and mouse brain (f), cross-sectional axis orientation images of rat muscle (g), and mouse brain (h). Image size: 1.75 mm (x), 945  μm (z) for rat muscle, and 4.2 mm (x), 945  μm (z) for mouse brain.

Figure 7 demonstrates the imaging results from other birefringent tissues. The left and right panels depict representative images from a fresh rat leg muscle and a fixed mouse brain slice, respectively. The imaging data, which contain 800 A-lines and 700 B-lines, are projected onto the xy plane to produce the en-face images [Figs. 7(a) and 7(b)] using the retardance contrast. These images respectively highlight different groups of muscles and white matter content. Green arrows indicate the locations of B-lines whose cross-sectional images are displayed below. The reflectivity images in Figs. 7(c) and 7(d) clearly show tissue structures without aforementioned artifacts. With a 25-dB dynamic range, the muscle image exhibits a relatively weaker signal strength that is persistent within a longer penetration depth. By contrast, the brain image with a 30-dB dynamic range shows a stronger signal for the white matter and higher overall attenuation. Figures 7(e) and 7(f) present the corresponding phase retardance images on the same scale (0 deg to 90 deg). As indicated by dark colors, the isotropic or non-birefringence regions do not vary in retardance values. The selected cross-section of the brain contains the corpus callosum, which is birefringent due to myelinated axon fibers. The retardance image helps reliably distinguish the white matter and gray matter regions. This is supported by near-zero retardance values of the gray matter, considerable reflectivity levels for both gray matter and white matter, and reduction of white matter reflectivity with inclination angle of axon fibers.^27^ The axis orientation images in Figs. 7(e) and 7(f) demonstrate consistent alignment of the adjacent muscle fibers and directions of the axon fibers in different corpus callosum regions.

## 4 Discussion and Conclusion

The visible-light BD PS-OCT is built mainly with bulk optics whose critical components are assembled on a fiber bench to aid alignment and light coupling to PM fibers. In the present design, the PM fibers play a crucial role in interference formation and convenient transmission of detected light to the spectrometer. Alternatively, the interference could be formed in the spectrometer;^19^ however, including the PM fiber in the reference and sample paths would result in a relative axis orientation measure due to a phase offset caused by environmental factors. The unique design of the spectrometer enables parallel detection of four spectral lines on a single camera. For each PS-OCT channel, a pair of highly aligned spectral lines yields the BD operation that provides a consistent signal gain over the imaging depth range.

Ordinary BD operation decreases the background level (Fig. 3) and removes the auto-interference components (Fig. 5) in the imaging range. These can be better achieved by implementing software corrections to match the spectral profiles of lines A and B. For instance, the grating efficiency varies differently for orthogonal polarization states, inducing an imbalance between the line pairs A and B. Therefore, the residual imbalance can be removed by multiplying each spectral line with a curve that accounts for such factors before subtracting the lines for BD. The results presented here are without applying such curves; therefore, there is room for further improvement.

The camera we used has a minimum exposure time of 24  μs, but the readout time slows the acquisition speed. With vertical binning of 4 pixels, we outputted four spectral lines in 295  μs. This may be reduced to 105  μs by optimizing the acquisition software. An ideal camera for this system would output two or four lines at high rates for demanding applications, preferably with rectangular pixels for easy alignment and robust operation. In addition, the BD operation might be implemented on a camera that only outputs the BD line(s) via firmware processing, further increasing the acquisition speed.

A significant amount (∼95%) of the supercontinuum laser’s output is discarded, as relatively low light powers directed towards the sample (1.1 mW) and reference (0.15 mW) paths are sufficient. Although the setup allows adjustment of equal reference light levels for the PS-OCT channels, the non-polarizing beamsplitter negligibly lowers (5%) light collection for one of the channels (Ch1). To extend the applicability of this technique to work with low-power sources, the beamsplitter might be replaced by a circulator; however, this could limit spectral bandwidth and impact the axial resolution.

The BD technique is especially advantageous for imaging high-scattering tissues. This is illustrated in Fig. 5, which shows significant auto-interference terms of the tendon sample without BD application. Importantly, BD removes the auto-interference terms and allows imaging in the shallower and more sensitive imaging depths. Although we gained expected improvements in signal amplitude and common noise suppression, BD did not lower the noise background at deeper imaging depths. For example, as presented in Figs. 3(c) and 3(d), average noise levels between 550 and 600  μm ranges are −61.2  dB with BD and −64.3  dB without BD. These results, a 3-dB increase in noise and a 6-dB increase in signal are similar to our observation with the BD system we reported for a non-PS OCT, which utilized a near-infrared superluminescent diode for light emission.^19^ The results indicate that the supercontinuum laser operating at 312 MHz repetition rate does not yield a dominant intensity noise for our system that operates at 24  μs exposure time (7488 pulses). However, using a more advanced camera with a shorter exposure time can more effectively demonstrate the need for BD operation in future studies.

In conclusion, we demonstrate a spectral domain PS-OCT technique capable of performing BD over a wide spectral range (from 467 and 604 nm) using a single camera. The characterization data and imaging results present expected improvements in SNR and removal of common noise. Leveraging the advantages of operation with broadband visible light, the system offers high spatial resolution, increased birefringence sensitivity, and the possibility of gaining further information by analyzing the imaging data in a few neighboring spectral bands. These benefits make the new visible-light PS-OCT system advantageous for imaging birefringent tissues.

## Data Availability

The code and data used in this study are available from the corresponding author upon reasonable request.
